# Spectral photon-counting CT imaging of colorectal peritoneal metastases: initial experience in rats

**DOI:** 10.1038/s41598-020-70282-w

**Published:** 2020-08-07

**Authors:** Arnaud Thivolet, Salim Si-Mohamed, Pierre-Emmanuel Bonnot, Christophe Blanchet, Vahan Képénékian, Loïc Boussel, Philippe Douek, Pascal Rousset

**Affiliations:** 1 Radiology Department, Hospices Civils de Lyon, pavillon B, 5 Place d’Arsonval, 69003 Lyon, France; 2 EMR 3738, Oullins, France; 3 Université Lyon 1 Claude Bernard, Lyon, France; 4 INSA‐Lyon, UJM-Saint Etienne, CNRS, Inserm, CREATIS UMR 5220, U1206, Lyon, France; 5 Surgical Department, Hospices Civils de Lyon, Lyon, France; 6 Pathology Department, Hospices Civils de Lyon, Lyon, France

**Keywords:** Preclinical research, Surgical oncology, Cancer imaging, Cancer models, Colorectal cancer

## Abstract

Computed tomography imaging plays a major role in the preoperative assessment of tumor burden by providing an accurate mapping of the distribution of peritoneal metastases (PM). Spectral Photon Counting Computed Tomography (SPCCT) is an innovative imaging modality that could overcome the current limitations of conventional CT, offering not only better spatial resolution but also better contrast resolution by allowing the discrimination of multiple contrast agents. Based on this capability, we tested the feasibility of SPCCT in the detection of PM at different time of tumor growth in 16 rats inoculated with CC531 cells using dual-contrast injection protocols in two compartments (i.e. intravenous iodine and intraperitoneal gadolinium or the reverse protocol), compared to surgery. For all peritoneal regions and for both protocols, sensitivity was 69%, specificity was 100% and accuracy was 80%, and the correlation with surgical exploration was strong (*p* = 0.97; *p* = 0.0001). No significant difference was found in terms of diagnostic performance, quality of peritoneal opacification or diagnostic quality between the 2 injection protocols. We also showed poor vascularization of peritoneal metastases by measuring low concentrations of contrast agent in the largest lesions using SPCCT, which was confirmed by immunohistochemical analyses. In conclusion, SPCCT using dual-contrast agent injection protocols in 2 compartments is a promising imaging modality to assess the extent of PM in a rat model.

## Introduction

Peritoneal metastases (PM) are part of the natural history of most abdominal and gynecological malignancies and have long been considered as a terminal disease. Over the past decades, the development of cytoreductive surgery with or without intraoperative hyperthermal intraperitoneal chemotherapy (HIPEC) has led to an improvement in survival of selected patients with resectable disease^1-4^. For colorectal cancer, this strategy has significantly improved oncology outcomes^4,5^. The completeness of the cytoreductive surgery is a crucial endpoint and is directly associated with the extent and distribution of PM in the peritoneal cavity^6,7^. However, this aggressive treatment is associated with substantial morbidity (30–40%) and a mortality rate that is reported to range from 0–10%, resulting in a further drastic selection of patients in the pre-treatment work-up^8,9^. In this context, preoperative evaluation of the peritoneal tumor burden, although extremely challenging, is essential for the selection of eligible patients for curative treatment^10,11^.

Computed tomography (CT) remains the reference imaging modality to assess the extent of PM. Although magnetic resonance imaging (MRI) and positron emission tomography-CT (PET-CT) have good diagnostic performance in some PM etiologies, these modalities are mainly used as a complement to CT evaluation^10,12-17^. However, even though CT imaging offers high spatial resolution and fast acquisition time, its main drawback is its lack of contrast resolution, leading to a significant underestimation of the number PM and therefore their distribution. This is especially true for the detection of small lesions (sensitivity of 10% to 43% for lesions < 0.5 cm), particularly in regions with a low contrast between the PM and surrounding structures^18,19^. Therefore, current peritoneal imaging could benefit from the new and promising technology that is Spectral Photon Counting Computed Tomography (SPCCT). SPCCT is an X-ray imaging technology based on new detectors known as Photon-Counting Detectors (PCD). Contrary to conventional or dual energy CT, that are equipped with energy-integrating detectors, these detectors record the energy of incident photons in multiple energy bins, providing a better sampling of spectral information with more than 2 energy levels^20-22^. The great advantage of this improved spectral sampling is the ability to detect a specific attenuation effect, called the K-edge, allowing specific discrimination and quantification of multiple contrast^23-28^. In order to provide these capabilities, PCDs have to respond to a high counting rate and detection efficiency, resulting in a combination of technological strategies that may overcome some of the limitations of conventional CT. Multiple in vitro studies have demonstrated the improvement in spatial resolution (< 0.6 mm) and contrast resolution by using this new technology^29-32^, with a few recent in vivo studies conducted on animals and human^24,26,33-37^. In a previous study, Si-Mohamed et al. demonstrated that, in peritoneal cavity and abdominal organ imaging of healthy rats, SPCCT increased the spatial resolution leading to excellent visualization of small peritoneal structures, but also the spectral resolution by allowing the differentiation of 2 contrast agents injected in 2 different compartments, i.e. intraperitoneal (IP) and intravenous (IV) injections^33^. The latter capability offers a specific and quantitative analysis of the contrast agent biodistribution resulting in greater contrast-to-noise ratios of the structures analyzed in comparison to conventional CT images^33^.

The purpose of the present study was therefore to assess the capacity of SPCCT to detect PM of colorectal cancer in a rat model using dual-contrast agent injection protocols in 2 compartments (IV and IP).

## Materials and methods

All experiments were carried out in accordance with the European directive 2010/63/UE and the French decree 2013-118 on the protection of animals used for scientific purposes and were approved by our local ethic committee (“Comité d'éthique pour l'Expérimentation Animale Neurosciences Lyon”, CELYNE , Reference No APAFIS #9083-2017022815189044).

### Syngeneic animal model

#### CC531 cell culture

The colon adenocarcinoma cell line CC531 was induced from the colon of rats exposed to methylazoxymethanol^38^. These cells were obtained from CLS Cell Lines Service (Eppelheim, Germany). The tumor cells were cultured at 37 °C in a 5% CO_2_ humidified atmosphere in a Roswell Park Memorial Institute 1640 medium (RPMI 1640; Life Technologies, Courtaboeuf, France) with 10% fetal calf serum (FCS; Life Technologies), 100 μM of streptomycin, and 100 U/mL of penicillin. Cell suspensions with less than 10 passages after thawing were prepared by enzymatic detaching of CC531 cells with trypsin–EDTA solution (Life Technologies) at room temperature (20 °C). After centrifugation at 500 g for 10 min, cell concentrations were resuspended in RPMI 1640 to the required concentration of 2.5 × 10^6^ cells per mL. The viability of the cells was assessed with trypan blue (0.1%) exclusion.

#### Animals and housing

To induce PM, 20 CC531-syngeneic male WAG/Rij rats (10–12 weeks old, mean ± SD weight 275 ± 25 g) were obtained from Charles River Laboratories (l’Arbresle, France)^39^. Animals were accustomed to laboratory conditions for at least 1 week before experimental use and housed under clean, non-sterile standardized conditions (temperature 20–24 °C; relative humidity 50%-60%, 12 h light/12 h dark) in filter-topped cages (3 rats per cage).

#### Induction of peritoneal carcinomatosis

Cell suspensions were prepared less than 1 h before the injection. Rats were anaesthetized with isoflurane (4% for induction, 1.5–2.5% for maintenance) in oxygen (1.5 L/min for induction, 0.6 L/min for maintenance) and an IP catheter (20G) was inserted in the right lower quadrant of the abdomen. Peritoneal metastases were induced using an IP injection of a single cell suspension containing 2.5 × 10^6^ cells/mL of colon carcinoma cell line CC531 in a volume of 2 mL. Sixteen rats were inoculated with CC531 cells, 4 rats were not initially inoculated and were only used in case of premature death (before the imaging evaluation date) to maintain homogeneity within groups.

### Spectral photon counting computed tomography (SPCCT)

The SPCCT (Philips Research, Haïfa, Israel) is a modified clinical CT that allows axial and helical scans over 360 degrees with a gantry rotation time of 1 s. SPCCT images were acquired at 100 mA tube current and 120 kVp tube voltage using a conventional X-ray tube emitting a spectrum of photons ranging from 30 to 120 keV. The detection system comprises 28 tiles of cadmium-zinc-telluride (CZT) sensors of 2 mm thickness and a pixel pitch of 500 μm × 500 μm, flip-chip bonded to Philips’ proprietary ChromAIX2 ASICs providing an in-plane field-of-view of 169 mm and a z-coverage at 2.5 mm in isocenter^40^. The scanner system is equipped with energy resolving PCDs allowing up to 5 consecutive energy bins at the detector level, which leads to a complete analysis of the transmitted spectrum through the animal. The photon counts are measured in one-sided bins by the scanner and then processed by subtraction into two-sided bins^20,40,41^. Spectral energy resolution (FWHM) is about 8% at 60 keV as shown previously^40^. The detailed detector response function is taken into account in the decomposition process. Iodine and gadolinium can be separated from a material decomposition process based on a maximum likelihood method.^24^ Of note, only the gadolinium separation is based on its K-edge energy, while the iodine separation is based on a 2 components model (water and iodine). Therefore, 2 energy bins were set at 50.2 keV (30–51, 51–64) to coincide with the K-edge energy of gadolinium, the extra energy bins were set to better managing the photon count rate (64–72, 72–85, 85–120). Further technical details are provided in previous studies^33,42^.

Conventional images and contrast material maps (i.e. iodine and gadolinium images) derived from SPCCT acquisitions were reconstructed on a voxel grid of 0.25 × 0.25 × 0.25 mm. A Gaussian filter of 2 pixels was applied on material decomposition iodine and gadolinium K-edge images for noise reduction, and contrast material overlay images were created using FIJI software (ImageJ)^39^.

### Experimental protocol

**Figure 1:**
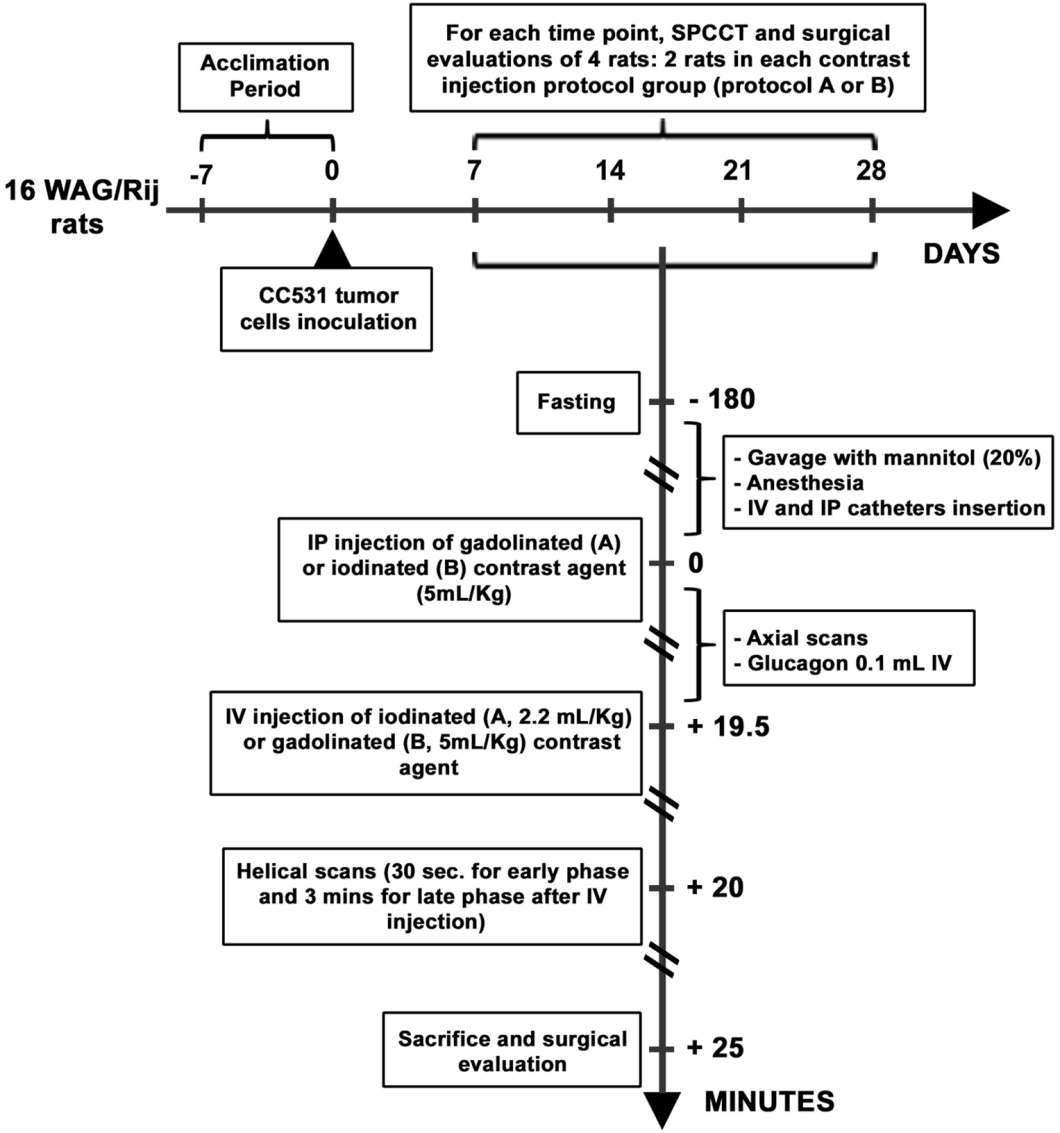
Experimental protocol.

Four timepoints were chosen to monitor tumor growth every 7 days after inoculation (D7, D14, D21, and D28) based on previous studies (Fig. 1)^43,44^.

#### Imaging protocol

Two contrast agents were used: a macrocyclic gadolinated one (Dotarem 0.5 mmol/mL; Guerbet, France) at a dose of 5 mL/kg for IV injections, and 20 mL/kg of a dilute solution (20%) for IP injections; an iodinated one (Xenetix 350 mg/mL; Bracco, France) at a dose of 2.2 mL/kg for IV injections and 20 mL/kg of a dilute solution (5%) for IP injections^33^.

The experimental plan was to explore at each timepoint 2 rats that received IP gadolinium then IV iodine injections (Protocol A), and 2 other rats that received an inversed injection protocol, i.e. IP iodine then IV gadolinium injections (Protocol B).

Rats were fasted for 3 h before the experiment and a hyperosmolar agent (1.7 mL of 20% mannitol; B. Braun Medical, Boulogne-Billancourt, France) was administered by oral gavage to improve small bowel analysis. Animals were anaesthetized with isoflurane (4% for induction, 1.5–2.5% for maintenance) in oxygen (1.5 L/min for induction, 0.6 L/min for maintenance) and monitored with a pulse oximeter and a heart-rate sensor during preparation and all acquisitions. A tail vein (22G) and an IP catheter (20G) were inserted for contrast injections. The IP injections were performed in the right lower quadrant of the abdomen and animals were installed in the ventral position on a small animal bed^45^. An intravenous injection of anti-peristaltic agent (0.1 mL of GlucaGen, glucagon, 1 mg/mL; Novo Nordisk, Chartres, France) was performed 5 min before the first helical acquisition. Blood glucose level was checked before glucagon injection and at the end of the experiment.

The imaging protocol included 3 axial slices at different levels of the abdomen immediately after IP injection of a contrast agent (iodine or gadolinium) to check the correct location of the IP contrast agent. Two helical acquisitions were then performed throughout the abdominopelvic region at 30 s (designated as the early phase) and 3 min (designated as the late phase) after IV injection (i.e. respectively 20 and 22.5 min after IP injection).

#### Surgical examination

Following SPCCT imaging, rats were immediately sacrificed using an intracardiac injection of pentobarbital (Dolethal 200 mg/kg, Vetoquinol, Aartselaar, Belgium). Autopsies were performed by a surgeon (P.E.B., 2 years of experience in surgery for this animal model) blinded to SPCCT results. The largest peritoneal tumor nodule in 1 rat of each injection protocol and timepoint (n = 8) was removed and sent for further pathological analysis.

#### Pathological examination

Tumor samples collected during surgery were fixed for 48 h in formalin and embedded in paraffin. Four micron-thick sections were cut from the paraffin blocks and stained with hematoxylin-phloxine-saffron (HPS). Slides were read in consensus by 2 pathologists (S.I. and C.B., with respectively 25 and 7 years of experience), blinded to the date of evaluation and injection protocol, to confirm the diagnosis of PM. For each largest peritoneal tumor nodule (n = 8), microvessel density (MVD) was quantified using immunohistochemistry on the BenchMark XT platform (Ventana Medical System, Inc., Tucson, AZ, USA) using an anti-ERG antibody (species mouse, clone 9FY, dilution 1/10, Zytomed, Berlin, Germany)^46^.

### Analyses

#### Surgical peritoneal cancer index

The surgical peritoneal cancer index (s-PCI), initially described by Jacquet and Sugarbaker in humans^47^, was adjusted in the present study to better suit the animal model (Supplementary Fig. 1). The abdominal and pelvic cavity was also divided by lines into 9 regions (regions 0 to 8) and the small bowel into 4 regions (regions 9 to 12), but only 3 small bowel regions were considered in the present study: proximal (region 9), intermediate (regions 10 and 11) and distal (region 12), due to the difficulty in differentiating distal jejunum and proximal ileum in rats. In order to maintain a s-PCI with a maximum score of 39, the same score value was assigned to regions 10 and 11. The peritoneal region corresponding to the cecum was also modified due to its more frequent location in the left iliac fossa in rats (region 5)^45^. During autopsies, the lesion size (LS) of the largest peritoneal implant for each region (longest diameter) was scored from 0 to 3 as described in a previous study (LS-0: no implants visible, LS-1: implants ≤ 2 mm, LS-2: implants > 2 mm and ≤ 5 mm, LS-3: implants > 5 mm)^48^. In case of confluent disease (region being coated by tumor) or tissue adhesions, the region was rated LS-3. The sum of LS scores per region produced a s-PCI between 0 and 39 (Supplementary Fig. 1).

#### Radiological analysis

SPCCT images of each rat were evaluated in consensus by 2 radiologists blinded to timepoints and surgical results (P.R. and A.T., respectively 16 and 5 years of experience in digestive radiology).

##### Diagnostic quality score

For each region, a subjective diagnostic quality score was also given based on the presence or absence of artifacts caused by respiratory motion and/or intestinal peristalsis, using the following score of diagnostic evaluation: 1: not possible; 2: acceptable; 3: good; 4: excellent.

##### Peritoneal opacification index

The quantity and distribution of the IP contrast agent was assessed on overlay images using a peritoneal opacification index (POI) by evaluating the degree of opacification in each region (0: none; 1: few; 2: correct; 3: good) as described in a previous study^33^. The sum of the opacification scores per region produced a POI between 0 and 39 (Supplementary Fig. 1).

##### Radiological peritoneal cancer index

Analysis of PM distribution was performed by combining the early and late acquisition phases on conventional and contrast material overlay images. The radiological PCI (r-PCI) was calculated with the same method as described for the s-PCI (Supplementary Fig. 1). Only unequivocal lesions of PM were reported such as the presence of nodules, pathological thick or pseudo nodular enhancement of the parietal or visceral peritoneum and pseudo nodular or nodular fat stranding in fat areas. Fat stranding was scored from 1 to 3 (1: mild, 2: intermediate, 3: diffuse, mass-like or omental cake), and the presence of a pathologic peritoneum enhancement without measurable nodular portion was scored 1.

##### Contrast agent concentrations in peritoneal metastases

On early and late-phase images, iodine and gadolinium concentrations were measured in the largest peritoneal tumor nodule, in omental cake (if present), and in the central part of the liver (excluding vessels, potential liver metastases, and interlobal IP contrast material) as a reference, by manually drawing regions of interests (ROI) using FIJI software (ImageJ)^39^. ROIs were standardized (3.3 mm diameter spherical ROI, 136 pixels), placed on SPCCT images and automatically copied onto the contrast material maps.

In case of artifacts that may interfere with measurements, such as ring artifacts or beam hardening artifacts related to excessive concentration of contrast agent (particularly for lesions near the bladder), another large target lesion was chosen. Lesions smaller than ROI (< 3.3 mm) were also excluded.

Concentrations of contrast agents are presented as mean ± standard deviation (SD) values in mg/mL.

##### Pathological analysis

For each selected rat (n = 8), the MVD was assessed in the largest peritoneal tumor nodule and in the peritumoral adipose tissue at × 200 magnification. Four fields containing the greatest number of vessels were selected in the tumor tissue (2 in the central region and 2 in the peripheral region of the tumor) and 2 fields in the surrounding normal peritoneal adipose tissue for comparison. The mean value of each region was used for further analysis and expressed as the number of vessels per field at magnification of × 200.

### Statistical analysis

Comparison between protocol A and B for POI and diagnostic quality scores, for each region separately and at the rat level, was performed using the Mann–Whitney U-test. Correlation and comparison between the r-PCI and s-PCI were assessed, respectively, using the Spearman’s rho correlation coefficient and the Wilcoxon matched-pairs signed rank test. Bland Altman plots were created by plotting the difference between s-PCI and r-PCI compared to the mean of s-PCI and r-PCI and the 95% confidence interval (mean ± 1.96 × SD). Sensitivity (Se), specificity (Sp), and accuracy (Acc) were calculated for both injection protocols (A + B) and for each one separately. Tumor depiction on CT per site was recorded independently of lesion size (tumor present versus absent). The Fisher's exact test was used to compare the diagnostic performance of protocol A versus protocol B and to compare the early assessment group (D7 + D14) versus the late assessment group (D21 + D28). The Wilcoxon matched-pairs signed rank test was also used to compare MVD in the tumor tissue versus its surrounding normal peritoneal adipose tissue. The null hypothesis was rejected for *p* < 0.05. Statistical analyses were performed using GraphPad Prism 7.0 (GraphPad Software, Inc., San Diego, CA, USA).

## Results

One rat taken for evaluation at D7 died at the moment of contrast agent injection and was replaced.

### Diagnostic quality score and peritoneal opacification index

No significant difference was found when comparing the POI and diagnostic quality score between protocol A and B by region and in total (Table 1).

**Table 1:** Diagnostic quality and peritoneal opacification index (POI) at the regional level.

#	Regions	Diagnostic quality score (Mean ± SD)			POI (Mean ± SD)			Protocols A + B (n = 16)	
		Protocol A (n = 8)	Protocol B (n = 8)	A versus B	Protocol A (n = 8)	Protocol B (n = 8)	A versus B	Diagnostic quality score	POI
				*p* values			*p* values	(Mean ± SD)	(Mean ± SD)
0	Central	3.13 (± 0.64)	3.38 (± 0.52)	0.674	2.63 (± 0.52)	2.88 (± 0.35)	0.569	3.25 (± 0.58)	2.75 (± 0.45)
1	RUQ	2.00 (± 0.53)	2.13 (± 0.64)	0.846	2.00 (± 0.76)	1.63 (± 0.52)	0.450	2.06 (± 0.57)	1.81 (± 0.66)
2	Epigastrium	2.00 (± 0.93)	1.88 (± 0.83)	0.826	2.25 (± 0.89)	1.50 (± 0.53)	0.117	1.94 (± 0.85)	1.88 (± 0.81)
3	LUQ	0.53 (± 3.88)	2.63 (± 1.06)	0.980	2.88 (± 0.35)	2.38 (± 0.74)	0.231	2.56 (± 0.81)	2.63 (± 0.62)
4	L Flank	3.88 (± 0.35)	3.13 (± 1.25)	0.200	3.00 (± 0)	2.63 (± 0.52)	0.200	3.50 (± 0.97)	2.81 (± 0.4)
5	LLQ	4.00 (± 0)	3.75 (± 0.71)	0.999	2.88 (± 0.35)	2.88 (± 0.35)	0.999	3.88 (± 0.50)	2.88 (± 0.34)
6	Pelvis	3.75 (± 0.71)	3.25 (± 0.89)	0.282	2.5 (± 0.76)	2.38 (± 0.74)	0.902	3.50 (± 0.82)	2.44 (± 0.73)
7	RLQ	4.00 (± 0)	4.00 (± 0)	0.999	2.88 (± 0.35)	3.00 (± 0)	0.999	4.00 (± 0)	2.94 (± 0.25)
8	R Flank	3.63 (± 0.52)	3.88 (± 0.35)	0.569	3.00 (± 0)	2.88 (± 0.35)	0.999	3.75 (± 0.45)	2.94 (± 0.25)
9	Proximal jejunum	1.88 (± 0.99)	1.63 (± 0.74)	0.695	2.38 (± 0.52)	2.25 (± 0.71)	0.999	1.75 (± 0.86)	2.31 (± 0.6)
10	Distal jejunum	2.75 (± 0.89)	2.50 (± 0.53)	0.730	2.75 (± 0.46)	2.50 (± 0.76)	0.713	2.63 (± 0.72)	2.63 (± 0.62)
11	Proximal ileum	2.75 (± 0.89)	2.50 (± 0.53)	0.730	2.75 (± 0.46)	2.50 (± 0.76)	0.713	2.63 (± 0.72)	2.63 (± 0.62)
12	Distal ileum	2.88 (± 0.83)	2.50 (± 0.93)	0.563	2.75 (± 0.46)	2.63 (± 0.74)	0.999	2.69 (± 0.87)	2.69 (± 0.6)
**All regions**		**Mean quality**	**Mean quality**	**p value**	**Total POI**	**Total POI**	**p value**	**Mean quality**	**Total POI**
		3.01 (± 0.99)	2.86 (± 1.03)	0.288	34.63 (± 2.62)	32.0 (± 4.21)	0.216	2.93 (± 0.76)	33.31 (± 3.65)

The mean ± SD diagnostic quality score was 2.93 ± 1.01 for protocols A + B (3.01 ± 0.99 for protocol A, and 2.86 ± 1.03 for protocol B; Table 1). There was no significant difference in the mean ± SD diagnostic quality score between the early (D7 + D14: 3.01 ± 0.80) and the late assessment groups (D21 + D28: 2.81 ± 0.76) (*p* = 0.158; Supplementary Table 1). For all protocols combined (A + B), the lowest diagnostic quality scores (mean score ≤ 2.00) were found in the epigastrium (region 2, score = 1 in 5 rats; 2 from protocol A and 3 from protocol B) and proximal jejunum (region 9, score = 1 in 7 rats; 3 from protocol A and 4 from protocol B); the highest diagnostic quality scores (mean score ≥ 3.00) were observed in the central region (region 0), left flank, left lower quadrant and pelvis (regions 4 to 6) and right flank (region 8); and up to 4 in right lower quadrant (region 7; Table 1).

The mean ± SD total POI score was 33.31 ± 3.65 (34.63 ± 2.62 for protocol A and 32.0 ± 4.21 for protocol B; Table 1). Mean total POI was 35.0 (± 2.21) in the early assessment groups (D7 + D14 groups) versus 31.6 (± 4.11) in the late assessment groups (D21 + D28 groups, *p* = 0.0067; Supplementary Table 1). The lowest mean POI were found in the right upper quadrant (region 1: 1.81 ± 0.66 for protocols A + B; 2.00 ± 0.76 for protocol A; 1.63 ± 0.52 for protocol B) and epigastrium (region 2: 1.88 ± 0.81 for protocols A + B; 2.25 ± 0.89 for protocol A; 1.50 ± 0.53 for protocol B); the highest POIs were found in right lower quadrant (region 7: 2.94 ± 0.25 for protocols A + B; 2.88 ± 0.35 for protocol A; 3.00 ± 0 for protocol B) and right flank (region 8: 2.94 ± 0.25 for protocols A + B; 3.00 ± 0 for protocol A; 2.88 ± 0.35 for protocol B, Table 1).

### Diagnostic performance

**Figure 2:**
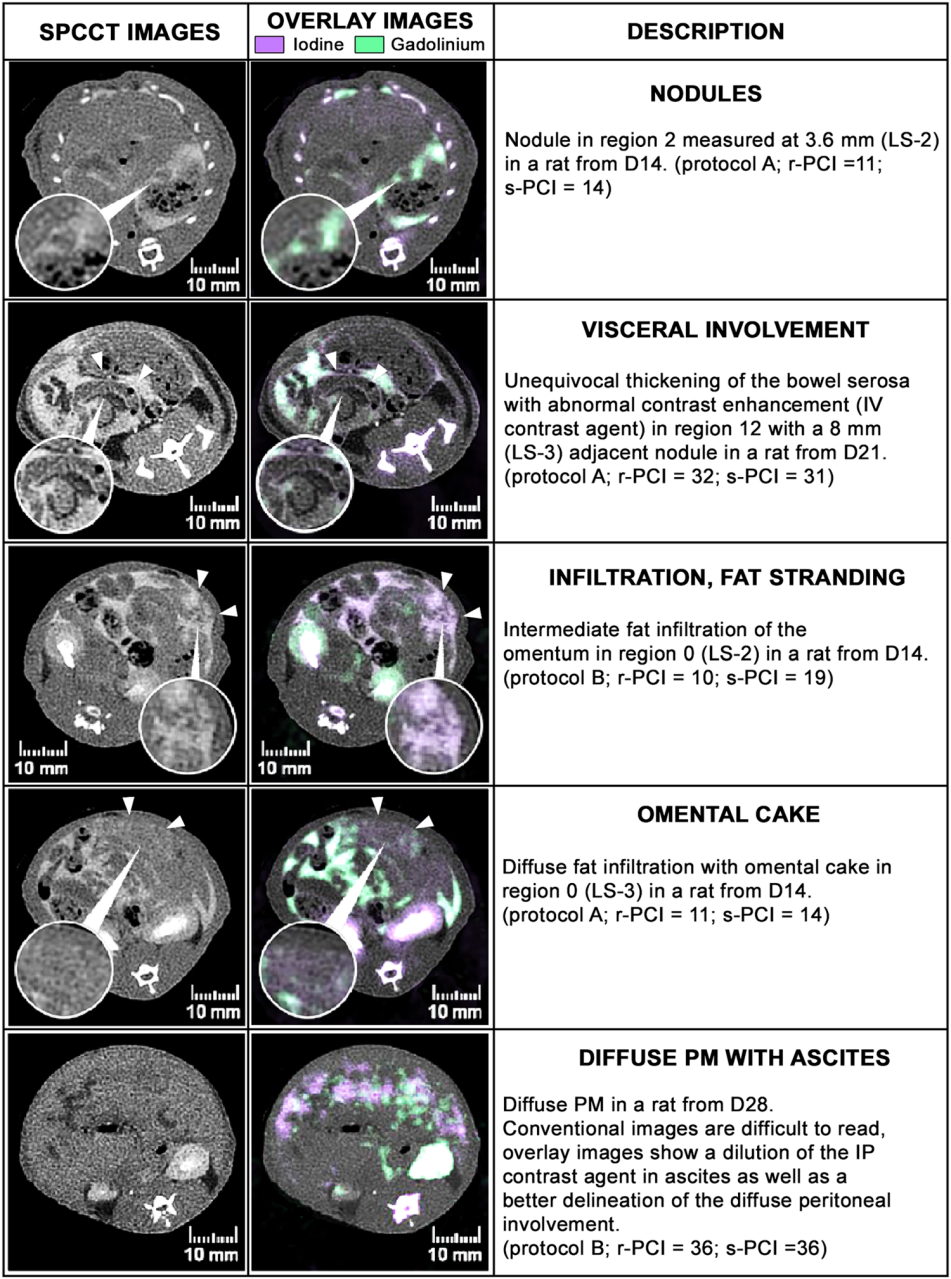
Illustration of SPCCT features of peritoneal metastases (PM) used to assess the radiological peritoneal cancer index (r-PCI). (LS: lesion size; s-PCI: surgical peritoneal cancer index).

Illustration of SPCCT features of PM used to assess the r-PCI are summarized in the Fig. 2.

On SPCCT, PM were found in 14 rats and 2 rats (12.5%; from D21 group) were free from PM; this was confirmed at surgery (Se = 100%, S*p* = 100%).

**Figure 3:**
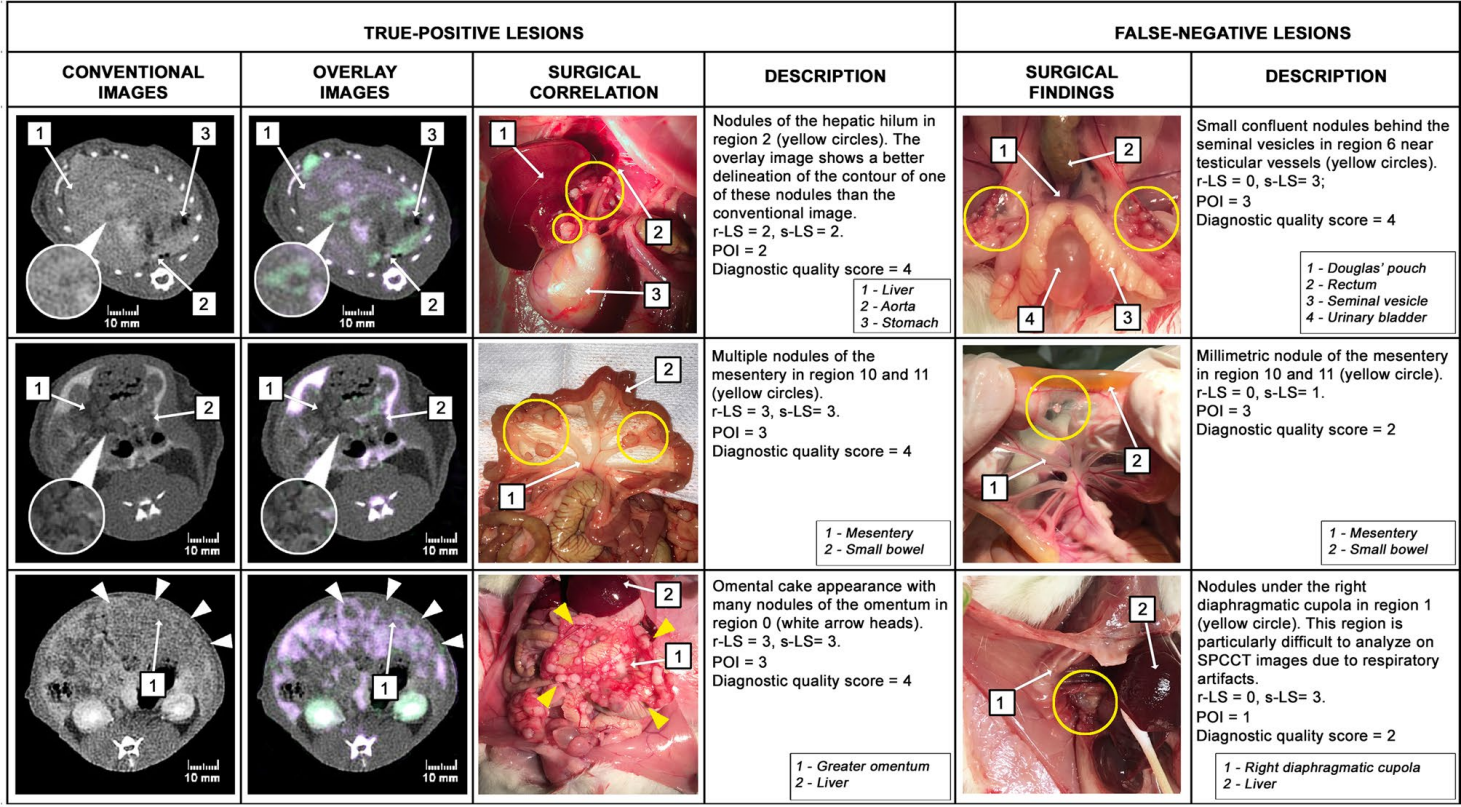
Examples of true-positive and false-negative lesions on SPCCT with surgical correlation (r-LS: radiological lesion size; s-LS: surgical lesion size; POI: peritoneal opacification index of the region).

Among all rats, PM were found in 135 regions at surgery (64 for protocol A and 71 for protocol B). Radiologists correctly depicted peritoneal lesions in 93 regions (42 for protocol A and 51 for protocol B); there were 42 false-negative regions (22 for protocol A and 20 for protocol B), 73 true-negative regions (40 for protocol A and 33 for protocol B) and no false-positive region (Fig. 3).

Overall, Se was 69%, Sp was 100%, and Acc was 80% (for protocol A: Se = 66%, Sp = 100%, Acc = 79%; protocol B: Se = 72%, Sp = 100%, Acc = 81%; Table 2). There was no significant difference in the Se of protocol A and B (*p* = 0.265), but a significant difference between the Se of early (D7 + D14: 56%) and the late assessment groups (D21 + D28: 80%, *p* = 0.008; Supplementary Table 1).

**Table 2:** SPCCT diagnostic performance for the detection of peritoneal metastases (TP: true-positive; FP: false-positive; TN: true-negative; FN: false-negative; Se: sensitivity; Sp: specificity; Acc: accuracy).

#	Regions	Protocols A + B							Protocol A							Protocol B						
		TP	FP	TN	FN	Se (%)	Sp (%)	Acc (%)	TP	FP	TN	FN	Se (%)	Sp (%)	Acc (%)	TP	FP	TN	FN	Se (%)	Sp (%)	Acc (%)
0	Central	11	0	2	3	79	100	81	5	0	1	2	71	100	75	6	0	1	1	86	100	88
1	RUQ	3	0	7	6	33	100	63	1	0	4	3	25	100	63	2	0	3	3	40	100	63
2	Epigastrium	8	0	3	5	62	100	69	4	0	2	2	67	100	75	4	0	1	3	57	100	63
3	LUQ	6	0	6	4	60	100	75	3	0	3	2	60	100	75	3	0	3	2	60	100	75
4	L Flank	2	0	10	4	33	100	75	1	0	5	2	33	100	75	1	0	5	2	33	100	75
5	LLQ	6	0	8	2	75	100	88	3	0	5	0	100	100	100	3	0	3	2	60	100	75
6	Pelvis	8	0	7	1	89	100	94	5	0	3	0	100	100	100	3	0	4	1	75	100	88
7	RLQ	9	0	6	1	90	100	94	5	0	3	0	100	100	100	4	0	3	1	80	100	88
8	R Flank	11	0	4	1	92	100	94	5	0	3	0	100	100	100	6	0	1	1	86	100	88
9	Proximal Jejunum	6	0	4	6	50	100	63	2	0	3	3	40	100	63	4	0	1	3	57	100	63
10	Distal jejunum	8	0	5	3	73	100	81	3	0	2	3	50	100	63	5	0	3	0	100	100	100
11	Proximal ileum	8	0	5	3	73	100	81	3	0	2	3	50	100	63	5	0	3	0	100	100	100
12	Distal ileum	7	0	6	3	70	100	81	2	0	4	2	50	100	75	5	0	2	1	83	100	88
**Total**		93	0	73	42	69	100	80	42	0	40	22	66	100	79	51	0	33	20	72	100	81

At the regional-level, lowest Se were found in the right upper quadrant (region 1: 33% for protocols A + B; 25% for protocol A; 40% for protocol B), left flank (region 4: 33% for protocols A + B; 33% for protocol A; 33% for protocol B) and proximal jejunum (region 9: 50% for protocols A + B, 40% for protocol A; 57% for protocol B); the highest Se were found in the right flank (region 8: 92% for protocols A + B; 100% for protocol A; 86% for protocol B), right lower quadrant (region 7: 90% for protocols A + B; 100% for protocol A; 80% for protocol B), and pelvis (region 6: 89% for protocols A + B; 100% for protocol A; 75% for protocol B, Table 2).

**Figure 4:**
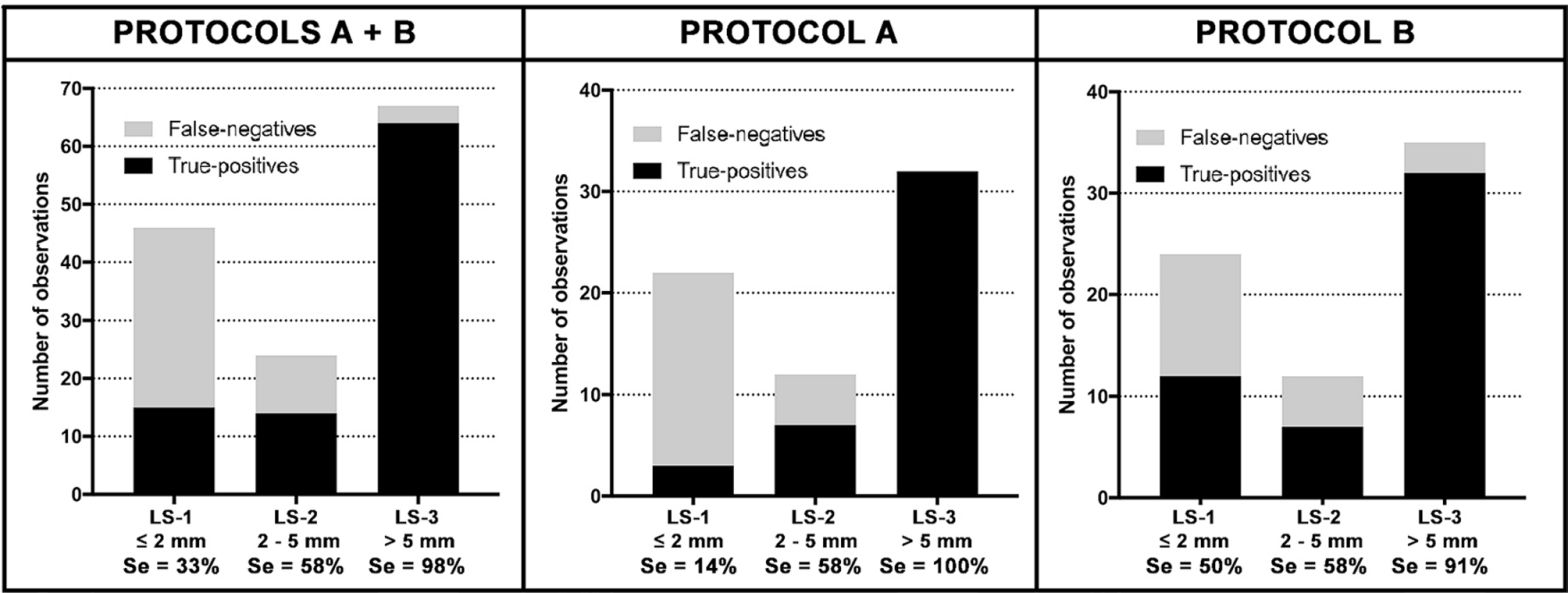
SPCCT sensitivity (Se) according to lesion size (LS).

The proportion of false negatives decreased with increasing lesion size (Fig. 4). Very small nodules (LS-1) were detected with a Se of 33% (14% for protocol A and 50% for protocol B) and 58% for LS-2 (58% for protocol A and 58% for protocol B). For lesions ≤ 5 mm (LS-1 and LS-2), Se were 41% for protocols A + B, 29% for protocol A and 53% for protocol B. The detection of larger or confluent lesions (LS-3) had a Se of 98% (100% for protocol A and 91% for protocol B).

### Correlation between surgical and radiological peritoneal cancer index

**Figure 5:**
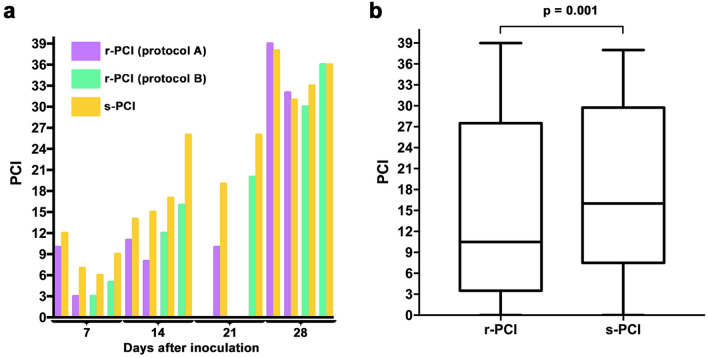
(**a**) Comparison of radiological (r-PCI) and surgical peritoneal cancer index (s-PCI) for each rat according to timepoint and injection protocol. (**b**) Box-and-whisker plots of the distribution of r-PCI and s-PCI for all protocols (A + B).

The median (range) s-PCI was 16 (0–38) and that of r-PCI was 10.5 (0–39); there was a significant underestimation of the s-PCI by the r-PCI (*p* = 0.001; Fig. 5). Se was 31% for low PCI (≤ 10), 43% for intermediate PCI (> 10 and ≤ 20) and 84% for high PCI (> 20; Supplementary Fig. 2). Distribution of peritoneal implants according to LS, as found during radiological and surgical evaluation, is summarized by region in Supplementary Fig. 3.

There was a very strong, significant correlation between r-PCI and s-PCI; Spearman’s correlation coefficients: ρ = 0.97 (*p* = 0.0001) for protocols A and B; ρ = 0.85 (*p* < 0.05) for protocol A; ρ = 0.99 (*p* = 0.0001) for protocol B.

According to Bland–Altman plots, for protocol A + B the bias was 3.4 and the 95% limits of agreement were − 3.3 to − 10.1; for protocol A the bias was 2.9 and the 95% limits of agreement were − 4.3 to 10.1; for protocol B the bias was 3.88 and the 95% limits of agreement were − 2.5 to 10.3 (Supplementary Fig. 4).

### Contrast agent concentrations and microvessel density quantification in peritoneal metastases

**Figure 6:**
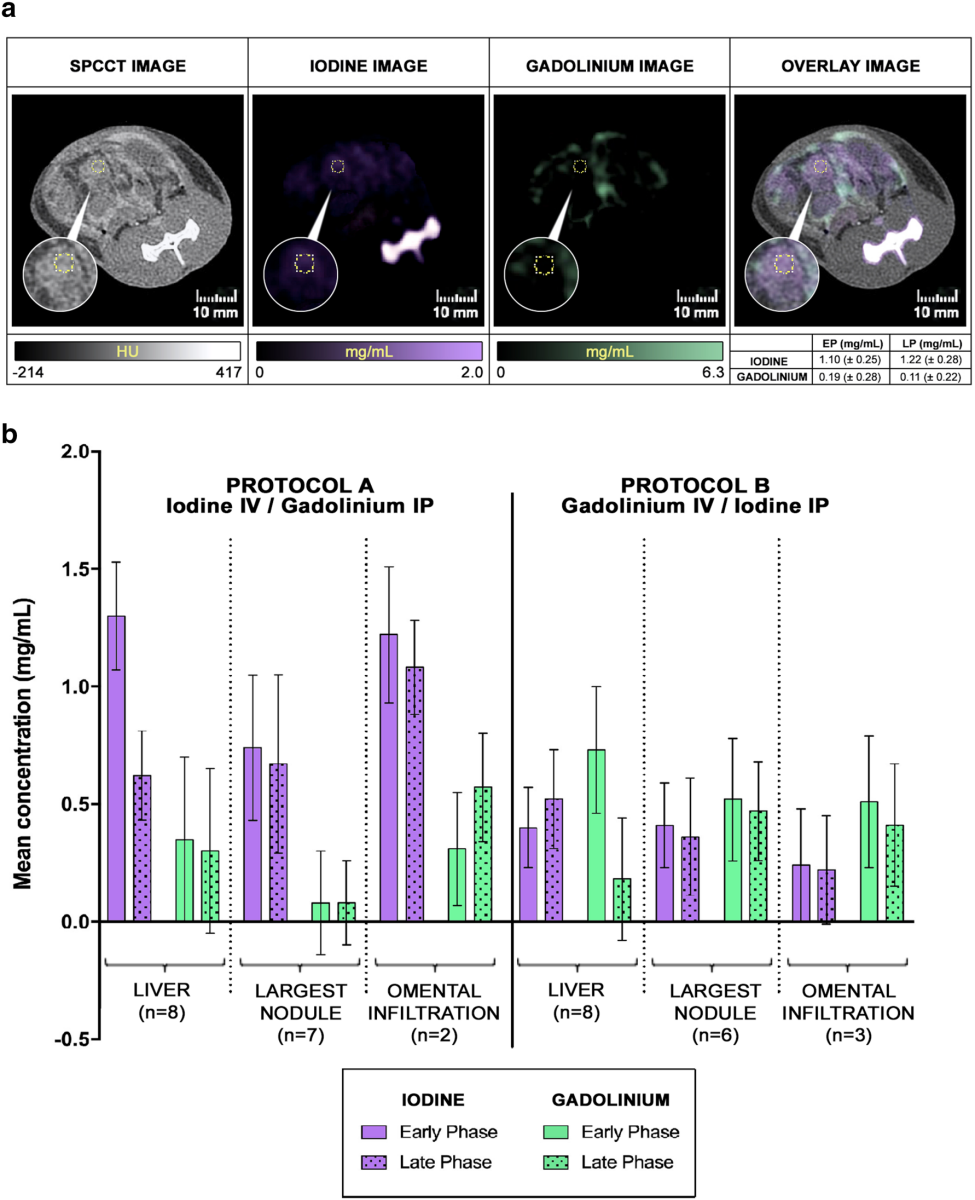
(**A**) Example of contrast agent concentration measurements in a peritoneal nodule in regions 10–11 (EP: Early phase, LP: Late Phase). (**B**) Concentrations of contrast agents measured on early and late-phase contrast material maps in the largest peritoneal tumor nodule, liver, and omental cake (if present). Data are presented as mean ± standard deviation of mean concentration.

A rather large peritoneal tumor nodule (i.e. > 3.3 mm in diameter) has been identified in 13 of the 14 rats with PM (n = 7 in protocol A and n = 6 in protocol B). The mean ± SD concentrations (IV and IP) measured in these tumor nodules on early and late-phase images were low in both protocols (0.74 ± 0.31 and 0.67 ± 0.38 mg/mL of iodine and 0.08 ± 0.22 and 0.08 ± 0.18 mg/mL of gadolinium in protocol A; 0.41 ± 0.18 and 0.36 ± 0.25 mg/mL of iodine and 0.52 ± 0.26 and 0.47 ± 0.21 mg/mL of gadolinium in protocol B); there were higher concentrations of IV contrast agent in protocol A (iodine) than in protocol B (gadolinium). Concentrations were higher for omental cake (1.22 ± 0.29 and 1.08 ± 0.20 mg/mL of iodine and 0.31 ± 0.24 and 0.57 ± 0.23 mg/mL of gadolinium in protocol A; 0.24 ± 0.24 and 0.22 ± 0.23 mg/mL of iodine and 0.51 ± 0.28 and 0.41 ± 0.26 mg/mL of gadolinium in protocol B). In comparison, mean ± SD concentrations of IV contrast agent in the liver were 1.30 ± 0.23 (early phase) and 0.62 ± 0.19 mg/mL (late phase) of iodine in protocol A, and 0.73 ± 0.27 (early phase) and 0.18 ± 0.26 mg/mL (late phase) of gadolinium in protocol B. Mean ± SD concentrations of IP contrast agent in the liver, reabsorbed from the peritoneal cavity, were 0.35 ± 0.35 (early phase) and 0.30 ± 0.35 mg/mL (late phase) of gadolinium in protocol A, and 0.40 ± 0.17 (early phase) and 0.52 ± 0.21 mg/mL (late phase) of iodine in protocol B **(**Fig. 6).

Immunohistochemistry found a mean ± SD 32.8 ± 25.5 vessels per field in the peripheral region of the peritoneal tumor nodule and 26.8 ± 20.6 in the central region; there was no significant difference with that found in peritumoral adipose tissue (29.2 ± 29.4, both comparisons *p* > 0.05; Supplementary Fig. 5).

## Discussion

In the present study, we demonstrated the capacity of a prototype SPCCT system to accurately detect PM in a rat model using dual-contrast agent injection protocols in the peritoneal and vascular compartments at different time of tumor growth. These results confirm in a disease model the high spatial and spectral contrast resolution previously reported in healthy rats by Si-Mohamed et al.^33^, which paves the way for application of PM SPCCT imaging in humans. It is of note that the diagnostic performance SPCCT was found to be in line with that reported for colorectal PM in humans using conventional CT, i.e. Se ranging, in function of lesion size and location, from 11 to 96% and Sp ranging from 49 to 100%^49^. However, in terms of absolute size, the Se of SPCCT in rats was much greater for lesions < 5 mm (LS-1 + LS-2) than the 11% Se for such lesions in humans (LS-1) reported by Koh et al. using conventional CT^19^. Despite this better performance, a low Se was found in some regions which could be explained by the presence of motion artifacts for regions 1 (respiratory movements) and 9 (bowel peristaltism); leading to low diagnostic quality scores and/or a reduced peritoneal opacification (reduced POI). Regarding the very low Se in region 4, this may be explained by the high proportion of small lesions (LS-1; < 2 mm). Sensitivities were greater for other regions, particularly in the intestinal regions (regions 9 to 12, range 50–73%), which is of importance as the detection of intestinal involvement has a great impact on the decision for complete cytoreductive surgery^50,51^. This is better than that found using conventional CT in humans; Esquivel et al. reported that these ranged from 45 to 52%^52^, and Koh et al. from 8 to 14%^19^. In addition, a PCI > 20 is often accepted as a contraindication to the cytoreductive surgery in human colorectal PM^53^. In the present study, the Se of the SPCCT was found to be greater than in humans for PCI > 20^52^, which could avoid unnecessary “open-close” procedures^53^.

No significant difference in diagnostic performance was found between the 2 injection protocols, which is probably explained by the absence of significant difference in either POI or diagnostic quality score. However, for both injection protocols the largest lesions were enhanced by IV contrast agent and could be detected by the readers on contrast material maps, as were those located in the greater omentum; the latter is in relation to the high vascularization of this region due to its remarkable angiogenic properties which also explains why it is a common location of PM^54^. For other lesions, the limited visualization on the contrast material maps was confirmed by the low concentrations of IV contrast agent. This may be explained by 2 factors: first, by the low vascularization of colorectal PM in this model, as confirmed by pathological analyses herein and their host organ (the peritoneum)^55,56^; second, by concentration values that may be below the detectability threshold of the prototype SPCCT as suggested in a previous in vitro study (~ 1 mg/mL)^29^. But current development in terms of advanced iterative reconstruction methods, such as one-step algorithms, might improve the detectability limit of contrast agents in peritoneal imaging^57^. It is also of note that the concentration of IV contrast agent in protocol B (gadolinium) was lower than in protocol A (iodine), which is likely to be explained by the difference in atom load injected IV between the protocols as with SPCCT technology only the number of atoms is detected (5 mL/kg of gadolinium, equivalent to ~ 392 mg of atoms, and 2.1 mL/kg of iodine, equivalent to ~ 735 mg of atoms)^34^. With regards to the IP contrast agents, low concentrations were measured in the PM which could be explained by the partial reabsorption by the peritoneum in the intravascular compartment, as previously suggested^58,59^.

The finding herein that only PMs of a certain size and/or in a certain location could be identified by readers on contrast material maps highlights the need to increase the spectral resolution of the SPCCT. Nevertheless, the spectral resolution was sufficient to improve the radiological analysis via the use of color overlay images (obtained by overlaying contrast agent maps with Hounsfield Unit images) and thus producing a spectral negative contrast of the peritoneal cavity, as demonstrated in previous studies^58,60^. Taken together, SPCCT appears to be a promising modality for the detection of small PM, with the potential for an earlier diagnosis and more accurate assessment of the extent of peritoneal disease.

Peritoneal imaging in small animals is challenging, and only a few studies have shown the potential of CT imaging in rodents (microCT) for the detection of PM, due to a poor soft tissue contrast resolution and time-consuming acquisitions, thus limiting the use of IV contrast agents^58^. Imaging techniques with high contrast resolution such as MRI, optical imaging and PET CT are more widely used in small animal^58,61-64^. However, the main limitation of these modalities is the lack of specificity which can lead to an overestimation of tumor burden^65^. Moreover, CT remains the reference imaging modality for the study of PM in humans, and in clinical practice MRI and PET CT are mostly used as a complement and the application of optical imaging is limited in humans^61,66^. SPCCT technology, combining high spatial and spectral contrast resolution, overcomes these limitations. In addition, with regard to small animal imaging, the presence of artifacts caused by intestinal peristalsis, heartbeats or respiratory movements must also be taken into account. The experimental protocol used herein overcomes these limitations; an antiperistaltic agent combined with fasting, and an oral injection of mannitol were used to reduce bowel peristalsis (which also reduced hardening artifacts), in addition, rats were in ventral position to reduce respiratory movements and thus their impact on the analysis of upper peritoneal regions (regions 1 to 3). Furthermore, 2 acquisitions (early and late phase) were made, allowing the interpretation of a region even if this was affected by motion artefacts on 1 of the acquisitions.

Based on this study, the SPCCT could have a promising role in the exploration of PM, but also in other applications in oncological imaging, particularly for the detection of more vascularized tumors or those located in another host organ (e.g. liver metastases)^67^. In the present study, no significant difference of diagnostic performance was found between the 2 injection protocols, probably because of the low contrast enhancement of peritoneal lesions observed, and because the injection protocol did not influence the POI and the diagnostic quality score. Regarding gadolinium contrast agent, our study adds to growing data demonstrating that it is a good candidate for SPCCT imaging as it allows good separation from body tissue and iodine based on its K-edge^29,33,36,68^. Taking into account that the amount of IV gadolinium of the protocol B was higher than the recommended clinical doses in MRI and the knowledge of gadolinium tissue deposition in brain and bone^69^, protocol A with IV appears to be more suitable for clinical research. Nevertheless, optimization of many parameters such as the concentrations of contrast agents (whatever iodine or gadolinium), their order and timing of injection should be evaluated and determined by future studies before human translation. Another issue will be the development of new contrast agents (nanoparticles) for K-edge imaging that may play a major role in future clinical applications of the SPCCT, such as a gadolinium-based nanoparticle (AGuIX) recently tested in human^26,68,70^.

This study has some limitations. First, the rat sample size was small and could have limited the validity of the results. Second, the rats used in this study were smaller than those used in a previous study (mean ± SD weight: 275 ± 25 g versus 510 ± 64 g)^33^, thus limiting the amount of contrast material injected reducing tumor enhancement, but also causing more motion artifacts in the upper mesocolic (1 to 3) and intestinal (9 to 12) regions which negatively affected the interpretation of images. However, the rat model evaluated is commonly used in surgical and oncological research for the development of new treatments (systemic or intraperitoneal). It allows a highly reproducible rate of tumor growth and spread that is similar to human PM^48,71^, and it is of note that the proportion of rats free from PM after inoculation was comparable to that reported elswehere^44,72^.

## Conclusion

SPCCT imaging using dual-contrast agent injection protocols in 2 compartments (intravascular and intraperitoneal) is a promising imaging modality to assess the extent of peritoneal metastases in a rat model.

## Supplementary information

Supplementary Information.
