# Supplementary material for: Spectral photon-counting CT imaging of colorectal peritoneal metastases: initial experience in rats
Source: Sci Rep. 2020 Aug 7;10:13394. doi: 10.1038/s41598-020-70282-w (PMC7414131; doi:10.1038/s41598-020-70282-w)
Supplement: Supplementary file 1 — Supplementary Information. [file 41598_2020_70282_MOESM1_ESM.docx]

**Spectral photon-counting CT imaging of colorectal peritoneal metastases: initial experience in rats**

**AUTHORS:**

Arnaud Thivolet^1,2,3*^, Salim Si-Mohamed^1,3,4^, Pierre-Emmanuel Bonnot^2,3,5^, Christophe Blanchet^6^, Vahan Képénékian^2,3,5^, Loïc Boussel^1,3,4^, Philippe Douek^1,3,4^, Pascal Rousset^1,2,3^

**AFFILIATION LIST:**

1 Hospices Civils de Lyon, Radiology department, Lyon, France

2 EMR 3738, Oullins, France

3 Université Lyon 1 Claude Bernard, Lyon, France

4 INSA*‐*Lyon, UJM-Saint Etienne, CNRS, Inserm, CREATIS UMR 5220, U1206, Lyon, France

5 Hospices Civils de Lyon, Surgical department, Lyon, France

6 Hospices Civils de Lyon, Pathology department, Lyon, France

**KEYWORDS:** multidetector computed tomography; spectral CT; dual-contrast imaging; K-edge imaging; photon-counting detectors; peritoneal carcinomatosis; peritoneal metastases; colorectal cancer

**CORRESPONDING AUTHOR:**

Arnaud Thivolet, M.D

Telephone number: +33 4 72 11 11 00

E-mail address: arnaud.thivolet@chu-lyon.fr

Address for correspondence: Hôpital Edouard Herriot, Service de radiologie - pavillon B, 5 Place d'Arsonval, 69003 Lyon France

**COMPETING INTERESTS**
The authors declare no competing interests.

**SUPPLEMENTAL MATERIAL**

**Figures**

**Supplementary Figure 1.** Schematic representation of regional segmentation in rats for the evaluation of peritoneal opacification index (POI) and peritoneal cancer index in rats (r-PCI: radiological peritoneal cancer index; s-PCI: surgical peritoneal cancer index).

**
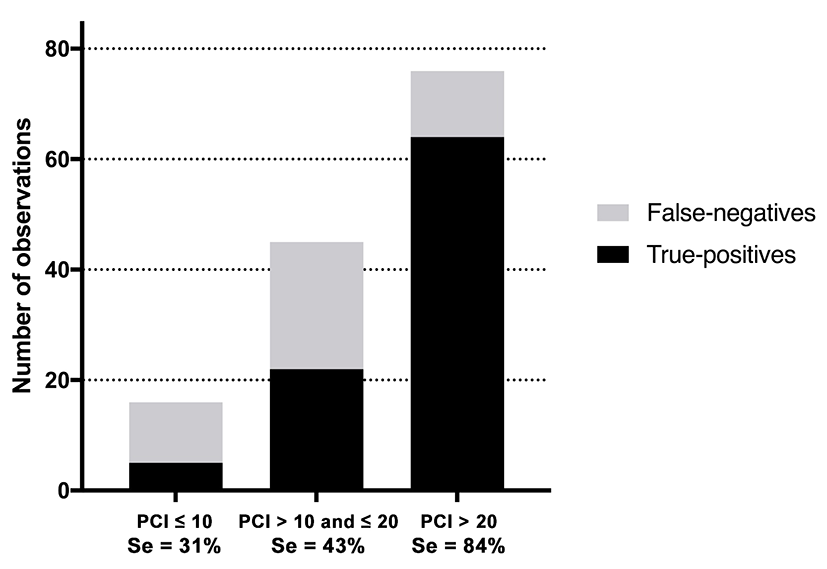
**

**Supplementary Figure 2.** Sensitivity (Se) according to surgical peritoneal cancer index (PCI) thresholds in all rats (protocols A+B).

**
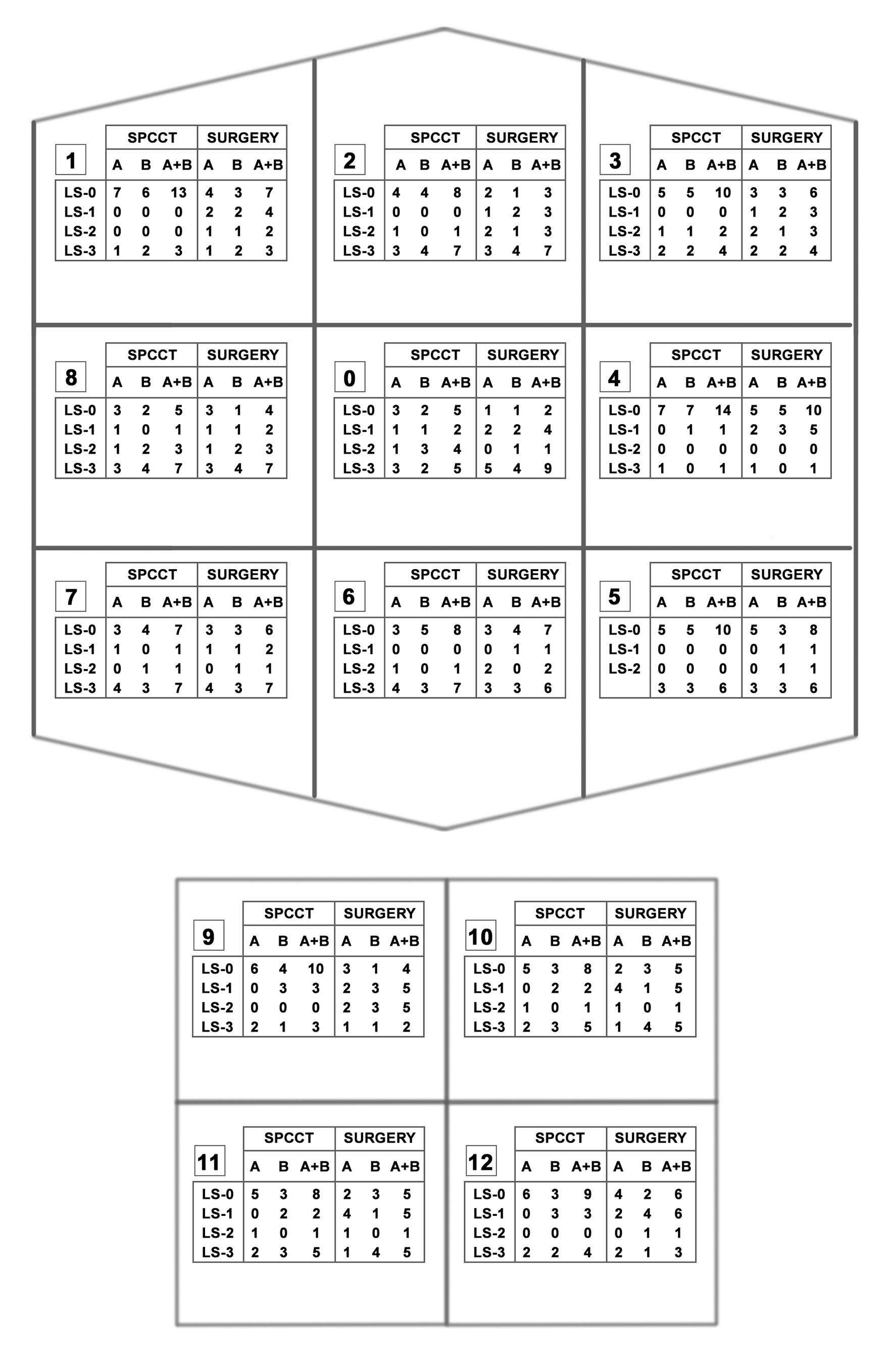
**

**Supplementary Figure 3.** Schematic representation of the peritoneal metastases distribution found on SPCCT and at surgery by region and lesion size (LS) for all rats (protocols A+B) and for each injection protocol (A or B).

**
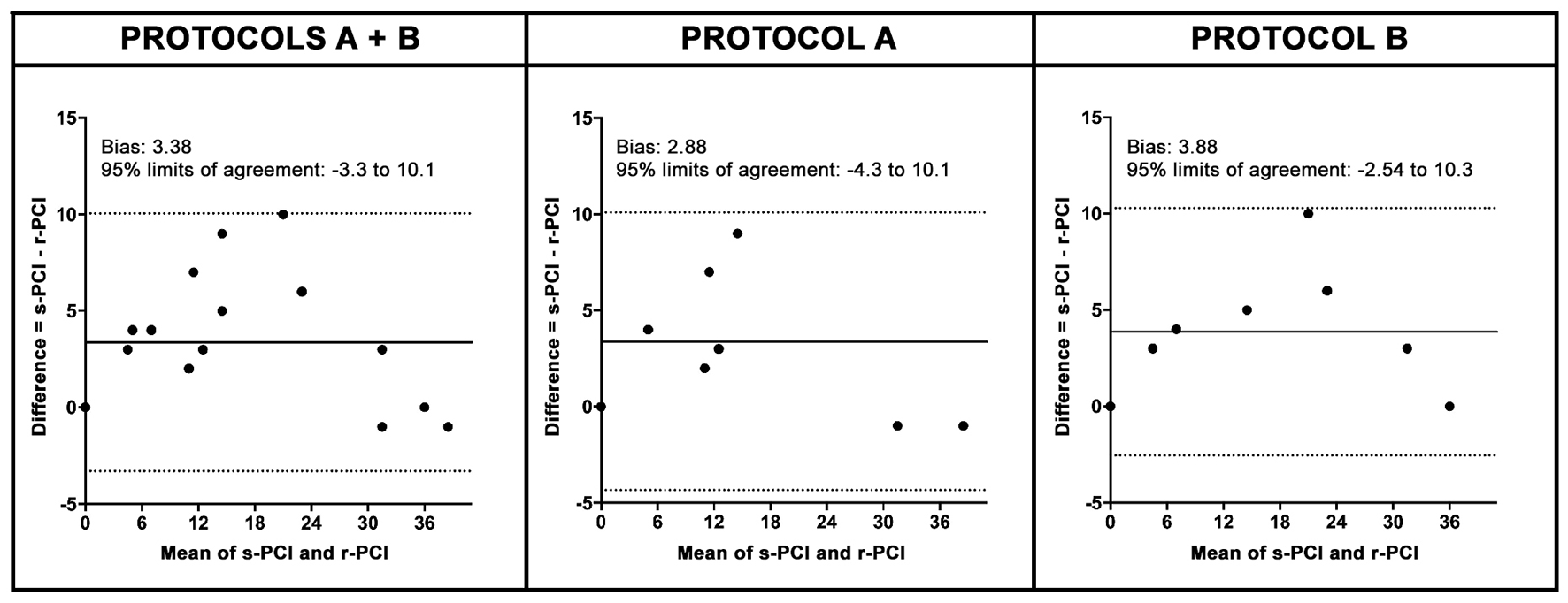
**

**Supplementary Figure 4.** Bland-Altman plots according to protocol.

**
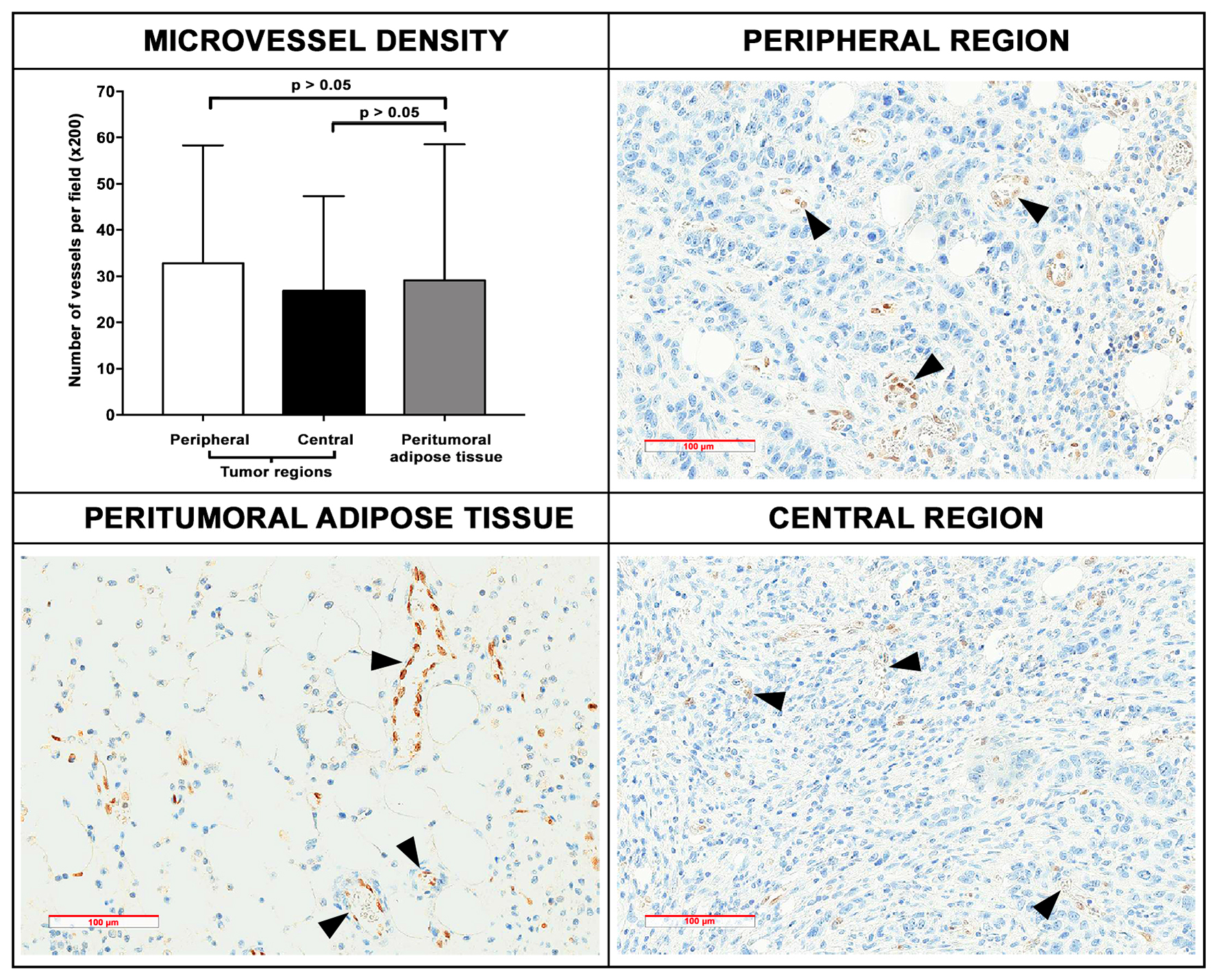
Supplementary Figure 5.** Quantification of ERG immunostaining: mean number of microvessels (+ standard deviation) per field at x200 magnification in the peripheral and central regions of the tumor tissue in comparison to the peritumoral adipose tissue. Positive staining of endothelial cells with ERG are shown as brown structures in each image (black head arrows: examples of vessels).

**Tables**

| **Groups** | **Diagnostic quality**  **score** (±SD) | **Mean POI**  (±SD) | **Mean PCI**  (±SD) | **TP** | **FP** | **TN** | **FN** | **Se** | **Sp** | **Acc** |
| --- | --- | --- | --- | --- | --- | --- | --- | --- | --- | --- |
| **D7 + D14** | 3.01 (±0.80) | 35.0 (±2.20) | 8.5 (±4.63) | 34 | 0 | 43 | 27 | 56% | 100% | 74% |
| **D21 + D28** | 2.81 (±0.76) | 31.6 (±4.1) | 20.9 (±15.8) | 59 | 0 | 30 | 15 | 80% | 100% | 86% |

**Supplementary Table 1.**  SPCCT analysis and diagnostic performance for early (D7 + D14) and late (D21 + D28) assessment groups (POI: peritoneal opacification index; PCI: surgical peritoneal cancer index; TP: true-positive; FP: false-positive; TN: true-negative; FN: false-negative; Se: sensitivity; Sp: specificity; Acc: accuracy)
